# The impact of the 2014 Ebola virus disease outbreak in Liberia on parent preferences for harsh discipline practices: a quasi-experimental, pre-post design

**DOI:** 10.1017/gmh.2017.24

**Published:** 2018-01-09

**Authors:** Eric Green, Rhea M Chase, John Zayzay, Amy Finnegan, Eve S. Puffer

**Affiliations:** 1 Duke University, Duke Global Health Institute, Box 90519, Durham, North Carolina 27708, USA; 2 Judge Baker Children's Center, Early Childhood Programs, Boston, Massachusetts, USA; 3 International Rescue Committee, Monrovia, Liberia; 4 Department of Psychology & Neuroscience, Duke University, Box 90086 417 Chapel Drive, Durham, NC 27708, USA

**Keywords:** Ebola virus disease, global mental health, Liberia parenting

## Abstract

**Background.:**

This paper uses data from a cohort of parents and guardians of young children living in Monrovia, Liberia collected before and after the 2014 outbreak of Ebola virus disease (EVD) to estimate the impact of EVD exposure on implicit preferences for harsh discipline. We hypothesized that parents exposed to EVD-related sickness or death would exhibit a stronger preference for harsh discipline practices compared with non-exposed parents.

**Methods.:**

The data for this analysis come from two survey rounds conducted in Liberia as part of an intervention trial of a behavioral parenting skills intervention. Following a baseline assessment of 201 enrolled parents in July 2014, all program and study activities were halted due to the outbreak of EVD. Following the EVD crisis, we conducted a tracking survey with parents who completed the baseline survey 12 months prior. In both rounds, we presented parents with 12 digital comic strips of a child misbehaving and asked them to indicate how they would react if they were the parent in the stories.

**Results.:**

Parents from households with reported EVD sickness or death became more ‘harsh’ (Glass's delta = 1.41) in their hypothetical decision-making compared with non-exposed parents, *t* (167)=−2.3, *p*  <  0.05. Parents from households that experienced EVD-related sickness or death not only reported significantly more household conflict and anxiety, but also reported that their child exhibited fewer difficulties.

**Conclusions.:**

Results support the need for family-based interventions, including strategies to help parents learn alternatives to harsh punishment.

## Background

The West African Ebola virus epidemic (2013–2016) was the deadliest outbreak of the Ebola virus disease (EVD) ever recorded (CDC, 2016). More than 11 000 people in the region died (WHO, 2016), and the impact on the economies of Guinea, Liberia, and Sierra Leone has been estimated at $2.8 billion dollars (World Bank, 2016). The effects of the epidemic will likely continue to be felt for some time. Survivors report a range of neurological and psychiatric sequelae (Howlett *et al.* 2017), and there are probably long-term psychosocial effects at the individual, community, and international levels (Van Bortel *et al.* 2016). In this paper, we examine the impact of the outbreak in Liberia on parents’ preferences for harsh discipline and wellbeing at the child, caregiver, and household levels. This study was conducted as part of a pilot randomized controlled trial of a parenting intervention for caregivers of young children living in Monrovia.

EVD is highly contagious, and control measures typically include case finding and isolation, contact tracing, supportive care at specialized Ebola treatment centers, and safe burial practices (WHO Ebola Response Team, 2016). In Liberia, many cases were first cared for by families within homes and community centers, exposing children and families to sickness and death as a result (Abramowitz *et al.* 2015; Kirsch *et al.* 2017). Survivors of EVD are no longer contagious, but recovery can be marked by stigma for patients and their families.

The impact of EVD exposure on family functioning has been compared with the psychosocial sequelae associated with HIV/AIDS (Davtyan *et al.* 2014; Hanson *et al.* 2016), another epidemic that provokes social stigma and rejection and can disrupt family functioning. Stigma and isolation of families who have experienced EVD can be barriers to recovery from trauma and return to normal family functioning (Rabelo *et al.* 2016). Consistent with this, in a study conducted by Hanson *et al.* (2016), EVD survivors and households whose members got sick or died reported higher levels of stress and community stigma than those within EVD hotspots who did not experience sickness or death within the household. Experiences of sickness and death were also associated with indicators of anxiety, depression, and PTSD (Mohammed *et al.* 2015; Hanson *et al.* 2016).

The West African Ebola virus epidemic began in December 2013 in Guinea, which shares a northern border with Liberia. Liberia registered its first cases of EVD in March 2014 in the northern county of Lofa. The outbreak moved south over the next few months, and the first patients died in Monrovia in June. By the time the baseline survey began on July 12, Liberia had at least 131 total cases and 84 deaths.

The situation was about to get much worse, but there was still widespread public denial about the outbreak. Nearly 2 weeks after the conclusion of the baseline survey, the Liberian government began a 10-day military quarantine of the pilot study community, resulting in a pause in all research and program activities (Onishi, 2014). Still, many seemed to deny that the EVD existed. The epidemic would go on to claim nearly 5000 lives in Liberia and 6500 lives in Sierra Leone and Guinea (WHO, 2016). The World Health Organization declared an end to the Liberia EVD outbreak in May 2015. While three more people would later die from the disease in Liberia, the country was returning to normal.

Just before restarting research and program activities following the EVD crisis, we conducted a brief tracking survey in June 2015 with parents who completed the baseline survey 12 months prior. The purpose of the tracking survey was to reconnect with enrolled parents, inform them about the pending launch of the program, and ask them about their EVD exposure and current situation. We use this longitudinal data to estimate the impact of EVD exposure on parents’ implicit preferences for harsh discipline.

## Methods

### Setting and participants

The pilot intervention was implemented by the International Rescue Committee (IRC) with adults living in Monrovia's West Point community. Monrovia is Liberia's capital and largest city, home to more than 1 million residents. West Point is a densely populated, low-income township in Monrovia. To be eligible to participate in the study, adults had to be at least 18 years old, live in West Point, and be a primary caregiver for a child (biological or otherwise) aged 3 or 4 years. If the adult was caring for more than one child in this age range, the parent was asked to identify one target child to participate in the child assessments that were part of the larger study (not described here).

### Procedures

**Fig. 1.**
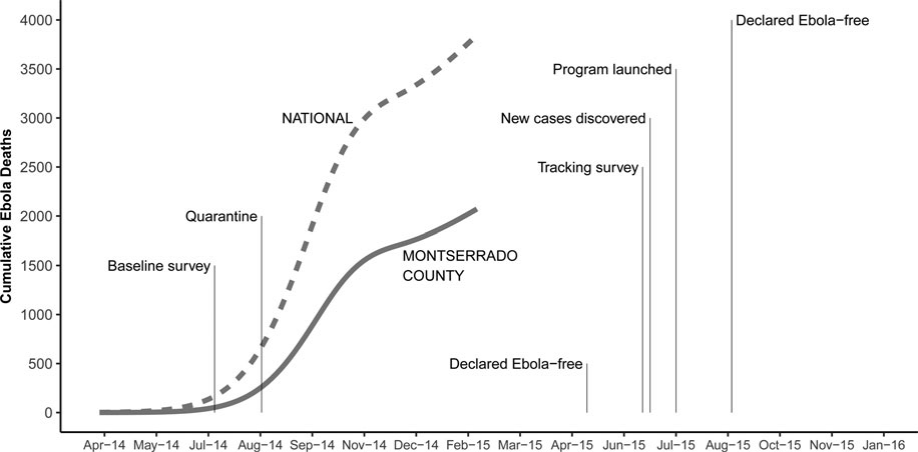
A timeline of the EVD outbreak and pilot study survey rounds.

In June 2014, we recruited 204 parents to participate in the pilot study. Staff from the IRC conducted informational meetings throughout the pilot community to raise awareness about the study and share eligibility guidelines. Interested parents were scheduled for a baseline survey if they met eligibility criteria. Then between 12 July 2014 and 7 August 2014, a team of trained Liberian enumerators attempted to visit the home of each eligible parent, obtain informed consent, and administer a baseline survey on an Android tablet; 201 parents completed the baseline survey. The following year – from 23 June 2015 through 27 August 2015 – the team attempted to locate these parents again to administer a post-EVD tracking survey prior to the start of the intervention. Efforts were made to survey parents who had moved away, even if they could no longer participate in the study (Fig. 1).

### Measures

All measures were created or adapted for the Liberian English vernacular and read aloud by a Liberian enumerator who recorded participant responses on a tablet computer.

#### Pre- and post-EVD measures of harsh discipline

The pre-EVD baseline and post-EVD tracking rounds both included an instrument modeled after discrete choice experiments. In this choice experiment, we presented parents with 12 digital comic strips of a young child misbehaving and asked them to indicate how they would react if they were the parent in the story. The purpose of designing this instrument was to reduce response bias given the potential for socially desirable responses to direct questions about discipline practices involving child maltreatment. See Green et al. (2017) for more details on the development and validation of this approach.

The comic strips had four attributes that varied on two or three levels each: (i) child gender (boy, girl); (ii) offense (spilling drink, whining, kicking parent); (iii) setting (home, market); and (iv) number of adults present (one, two). There were 24 possible permutations of these attributes and levels, but we used a fractional factorial design (orthogonal and balanced) and presented only 12 versions to each parent to limit responding fatigue. After viewing each story, participants indicated what they would do in the situation: time out, beat with an object, talk with the child, yell, or ignore. We classified yelling and beating as ‘harsh’ discipline (1; 0 if non-harsh) and summed across all 12 comic strips to create a harsh discipline preference score for each parent. Thus, raw scores could range from 0 (no endorsement of harsh discipline) to 12 (high endorsement of harsh discipline).

### Post-only measure of EVD exposure and correlates of exposure

#### EVD exposure

In the tracking survey conducted 10 months after the community was quarantined – only two months after Liberia was first declared ‘Ebola free’ – we asked parents about the health status of everyone listed in the household at the time of the baseline survey: ‘Did any of these people get sick with the Ebola virus disease?’ and ‘Did any of these people die after getting sick?’ Participants who responded ‘yes’ to either question were coded as ‘1’ to make a dichotomous variable of EVD exposure.

#### Household conflict and EVD-related hardship

We showed participants a picture of a 10-step ladder and asked them to tell us on which step their household was situated with respect to EVD-related hardship and household quarrels. EVD-related hardship (struggling) was assessed with the item: ‘Imagine a ladder with 10 steps. On the bottom step, Step 1, are households who survived [the outbreak of] Ebola but life is hard for now. On the last step up, Step 10, are households who survived Ebola and are doing well today.’ Household quarrels (conflict) was assessed with the item: ‘Imagine the 10-step ladder again. On Step 1 are households whose members are unhappy together and quarrel often. On Step 10 are households whose members live in harmony with each other.’ Responses to both items were reverse coded so that higher scores reflect worse outcomes.

#### Parent anxiety

We included a single-item indicator of parent anxiety (also reverse coded): ‘Imagine the 10-step ladder again. On Step 1 is a person who has no happiness or worries all the time. On Step 10 is a person who has much happiness and no worries.’

#### Child wellbeing

Parents completed the 25-item Strengths and Difficulties Questionnaire (SDQ; Goodman, 1997), a measure of children's emotional and behavioral wellbeing. Parents rated statements about their child on a three-point scale: (0) ‘not true’, (1) ‘somewhat true', and (2) ‘certainly true’. Four subscales reflect domains of difficulty, including Emotional Symptoms, Conduct Problems, Hyperactivity/Inattention, and Peer Relationship Problems. These items were summed to generate a Total Difficulties score.

#### Economic status

Parents also answered standard questions from the 2013 Liberia Demographic and Health Survey about household assets, housing materials, and access to water and sanitation facilities (DHS VI; LISGIS *et al.* 2014). Wealth index values were constructed using published results of a principal components analysis of the 2013 Liberia DHS (DHS Program, 2017).

### Statistical analysis

We estimated the impact of EVD exposure on parents’ preferences for harsh punishment by comparing the change in parent's endorsement of harsh consequences from before to after the EVD outbreak by EVD exposure status. The same sample of parents completed the choice experiment just prior to the EVD outbreak and again during the tracking survey run approximately two months after Liberia was first declared ‘Ebola-free’. We calculated pre–post gain scores and compared these scores by EVD status using a two-sample *t* test. We also used logistic regression to examine the relationship between baseline demographic variables and EVD-related illness and death, and ordinary least-squares regression to estimate the association between EVD exposure and wellbeing of households, parents, and children.

### Ethical review

This study protocol was reviewed and approved by the University of Liberia Pacific Institute for Research & Evaluation Institutional Review Board and the Duke University Institutional Review Board. All participants provided written informed consent to participate.

## Results

### Participant characteristics

A total of 201 parents completed the baseline survey, and 185 of these parents completed the post-EVD tracking survey. Of the 16 parents who did not complete the tracking survey, 14 moved out of the community, one died, and one could not be located. Participant characteristics are summarized in Table 1. We observed only minor differences by tracking survey completion status. On average, parents who did not complete the tracking survey were slightly more likely to be married or cohabiting with a partner compared to caregivers who completed the survey. Overall, the pilot study sample consisted of mostly female caregivers who were married with little formal education.

**Table 1.** Participant baseline characteristics

Variables	Baseline	Tracking survey	
		Found	Not found
*Parents*			
*N*	201	185	16
Mean Age (s.d.)	33.6 (10.2)	33.7 (10.4)	32.6 (8.3)
Female (%)	90.5	90.8	87.5
Completed primary (%)	5.5	5.4	6.2
Married or cohabiting (%)	75.6	74.6	87.5

### EVD exposure

According to the World Health Organization, there were 4506 confirmed, probable, and suspected cases of EVD in Montserrado County where Monrovia is located. With a population of 1.5 million residents in the county, this is a case rate of 0.30%. We found a case rate about six times higher among the household population of the study sample located during the tracking survey; 20 of 1099 household members got sick from EVD. Out of the original sample of 201 parents who completed the baseline survey, one died from EVD; an additional eight household members living with parents in the tracking sample also died. Overall 6.5% of households reportedly experienced EVD-related sickness or death. EVD illness or death was not significantly associated with household-level variables measured at baseline, such as economic status, water, and sanitation, or household size (see Table 2).

**Table 2.** Predictors of EVD sickness or death in the household

	Dependent variable:
	EVD in household
Wealth index	0.79 (0.20, 2.96)
Has improved sanitation	1.89 (0.38, 7.37)
Has improved water source	0.61 (0.14, 4.32)
Time to walk to water source (min)	1.03 (0.99, 1.07)
Treats drinking water	1.22 (0.34, 4.53)
Household size	1.12 (0.89, 1.37)
Constant	0.04** (0.003, 0.42)
EVD in household	6.5%
Observations	184

*p < 0.05,**p < 0.01, ***p < 0.001.

This table reports exponentiated coefficients (95% confidence intervals) from a logistic regression of EVD exposure on a set of household-level variables measured at baseline.

### Impact of EVD exposure on harsh punishment

On average, parents’ preference for harsh punishment decreased by 28.1% pre-EVD to post-EVD, from 4.7 (s.d.  =  3.1) to 3.4 (s.d.  =  3.0). However, exposure to EVD was associated with an increase in parent preferences for harsh punishment; parents from households with reported EVD sickness or death became more ‘harsh’ in their hypothetical decision-making (*M*  =  1.0, s.d.  =  4.0) compared with non-exposed parents (*M* = −1.5, s.d.  =  3.8), *t*(173) = −2.1, *p*  <  0.05. This is a standardized effect size (Glass's delta) of 1.38 standard deviations.

### Associations between EVD exposure and wellbeing

As shown in Table 3, parents from households that experienced EVD-related sickness or death not only reported significantly more household conflict and personal worries, but also reported that their child exhibited fewer difficulties. Children exposed to EVD sickness and death in the household had a 16.2% lower SDQ Total Difficulties score compared to children in households not exposed. As shown in online Supplementary Table A.1 in the Appendix, this effect appears to be driven by an increase in peer problems and, to a lesser extent, an increase in conduct problems among non-exposed children. There was not a significant relationship between EVD exposure and EVD-related hardship (struggles), suggesting that suffering was not limited to households that reported sickness or deaths.

**Table 3.** Associations between EVD exposure and wellbeing

		Dependent variable:			
		Struggling (household)	Conflict (household)	SDQ (child)	Anxiety (parent)
EVD in household		1.32 (1.07)	2.36*** (0.73)	−0.15** (0.07)	2.17* (1.11)
Female child				*−*0.05	
				(0.03)	
Child is 4				*−*0.10*** (0.04)	
Female parent					2.19**
					(1.02)
Parent age					0.02
					(0.03)
Constant		5.02*** (0.27)	1.22*** (0.19)	0.92*** (0.04)	1.07 (1.54)
Mean (s.d.) of Dep. Variable		5.1 (3.6)	1.4 (2.5)	0.8 (0.2)	3.9 (3.8)
Observations		184	183	185	184

*p *<* 0.05,**p *<* 0.01, ***p *<* 0.001.

This table reports the results of separate regressions of each dependent variable on an indicator of EVD in the household. The regression involving child SDQ controls for child gender and age at baseline, and the regression involving parent anxiety controls for parent gender and age at baseline.

## Discussion

This study makes a unique contribution to the literature on community- and family-level risk factors for violence against children. Results suggest that the experience of being closely affected by EVD through sickness or death could increase parents’ preference for harsh verbal or physical discipline. If these preferences were to translate into behavior, children in these households may be facing an elevated risk of maltreatment, potentially in addition to traumatic exposures associated with EVD. Exposed families also reported lower levels of overall family wellbeing, suggesting even broader patterns of negative interactions that can contribute to family violence and poor child outcomes.

Results are consistent with studies showing associations between stressors at the family and community levels and increases in child maltreatment (Cicchetti & Lynch, 1993). The effects of such stressors on parents’ mental health is one probable mediator, as parental stress is associated with harsher discipline practices (Maguire-Jack & Wang, 2016).

The finding that EVD exposure was associated with fewer emotional and behavioral symptoms among children was very unexpected. Given the potential traumatic experiences during the epidemic and the mental health problems often associated with family conflict and harsh discipline, we hypothesized results in the opposite direction. To explore potential reasons, it may be valuable first to consider situational factors that could influence parent report in such circumstances, such as potential decreases in awareness of children's emotions and behaviors when there are innumerable demands on parents’ time. It is also possible that children in exposed households experienced decreased, or different, demands and expectations given the changes to normal school and household activities; for instance, there may have been fewer opportunities for non-compliant behaviors during school closings.

Additionally, the definition of exposure should be considered, as even children not exposed to sickness or death likely experienced distress; all families in these communities faced the fear of sickness, disruptions to routines, resource constraints and, in some cases, temporary displacement. Children also may have experienced other potential traumas in their communities, such as directly viewing corpses, which has been associated with mental health symptoms (Mohammed *et al.* 2015; Hanson *et al.* 2016). Thus, EVD exposure measure in this study may not have captured the full range of ways in which epidemic-related experiences could affect child mental health. Future research with data on a broader range of stressors that are part of ‘exposure’ may indeed find links between specific stressors and emotional and behavioral outcomes. These are only examples of context-related considerations to explore in future studies.

Several limitations stem from the brief nature of this study. The tracking survey that was used to collect the ‘post’ EVD data was not part of the original study protocol. When we realized the need to re-connect with enrolled participants before resuming program activities following the outbreak, we also decided to obtain approval to expand the scope of the survey slightly to include questions about parenting behavior and wellbeing. With limited resources for an additional survey, we were unable to measure some constructs in as much depth as we would have liked. For instance, we were only able to ask parents one question about the degree to which they have worries. For this reason, we must avoid over-interpreting the results as conclusive evidence regarding the impact of EVD exposure on parental mental health. Similarly, we were not able to assess the full range of possible parenting and family variables, such as neglect and changes in family schedules, roles, and expectations. As discussed above, the definition and measurement of EVD exposure in this study were also limited to sickness and death, without expanding to include a broader range of epidemic-related stressors that could also affect child, parent, and family outcomes.

In conclusion, results suggest that family-based intervention approaches may be valuable in post-epidemic settings, including specific interventions to provide parents with alternative discipline strategies during times of elevated stress. Considering limited resources, findings suggest potential benefits of targeting behavioral parenting interventions for families who have been directly affected by an epidemic. Parent-level mental health interventions also likely have a place in the prevention of child maltreatment in these contexts. Results of the current study do not point toward the need for direct child mental health interventions, though one study should not lead to the conclusion that they are not needed. One possibility is that family-based interventions may be more indicated for young children whereas older children may exhibit more acute mental health symptoms that would respond best to a combination of family-and individual-level approaches.

## Supplementary material

For supplementary material accompanying this paper visit http://dx.doi.org/10.1017/gmh.2017.24. click here to view supplementary material

## References

[ref1] Abramowitz SA, McLean KE, McKune SL, Bardosh KL, Fallah M, Monger J, Tehoungue K, Omidian PA (2015). Community-centered responses to Ebola in urban Liberia: the view from below. PLoS Neglected Tropical Diseases 9, e0003706. 25856072 10.1371/journal.pntd.0003706 PMC4391876

[ref2] CDC (2016). Outbreaks chronology: Ebola virus disease. https://www.cdc.gov/vhf/ebola/outbreaks/history/chronology.html.

[ref3] Cicchetti D, Lynch M (1993). Toward an ecological/transactional model of community violence and child maltreatment: consequences for children's development. Psychiatry 56, 96–118. 8488217 10.1080/00332747.1993.11024624

[ref4] Davtyan M, Brown B, Folayan MO (2014). Addressing Ebola-related stigma: lessons learned from HIV/AIDS. Global Health Action 7: 1, 26058, DOI: 10.3402/gha.v7.26058. PMC4225220 25382685

[ref5] DHS Program (2017). Wealth index construction. http://www.dhsprogram.com/topics/wealth-index/Wealth-Index-Construction.cfm.

[ref6] Goodman R (1997). The strengths and difficulties questionnaire: a research note. Journal of Child Psychology and Psychiatry 38, 581–586. 9255702 10.1111/j.1469-7610.1997.tb01545.x

[ref19] Green EPD, Chase R, Zayzay J, Finnegan A, Puffer E (2017). A discrete choice task to measure preferences for harsh discipline among parents of young children. Retrieved from http://psyarxiv.com/egqjv.

[ref7] Hanson J, Decosimo A, Quinn M (2016). Diminished quality of life among women affected by Ebola. Journal of Social, Behavioral and Health Sciences 10, 112–133.

[ref8] Howlett P, Walder A, Lisk D, N'jai A, Lado M, Brown C, Sesay F, Semple M, Scott J (2017). Neurological and psychiatric manifestations of post Ebola syndrome in Sierra Leone. The Lancet 389, S48.

[ref9] Kirsch TD, Moseson H, Massaquoi M, Nyenswah TG, Goodermote R, Rodriguez-Barraquer I, Lessler J, Cumings D, Peters DH (2017). Impact of interventions and the incidence of Ebola virus disease in Liberia: implications for future epidemics. Health Policy and Planning 32, 205–214. 28207062 10.1093/heapol/czw113 PMC6279138

[ref10] LISGIS, Ministry of Health and Social Welfare/Liberia, National AIDS Control Program/Liberia, ICF International (2014). Liberia Demographic and Health Survey 2013. Technical Report, LISGIS and ICF International: Monrovia, Liberia.

[ref11] Maguire-Jack K, Wang X (2016). Pathways from neighborhood to neglect: the mediating effects of social support and parenting stress. Children and Youth Services Review 66, 28–34.

[ref12] Mohammed A, Sheikh TL, Gidado S, Poggensee G, Nguku P, Olayinka A, Ohuabunwo C, Waziri N, Shuaib F, Adeyemi J, Uzoma O, Ahmed A, Doherty F, Nyanti SB, Nzuki CK, Nasidi A, Oyemakinde A, Oguntimehin O, Abdussalam IA, Obiako RO (2015). An evaluation of psychological distress and social support of survivors and contacts of Ebola virus disease infection and their relatives in Lagos, Nigeria: a cross sectional study 2014. BMC Public Health 15. 10.1186/s12889-015-2167-6 PMC4550041 26307047

[ref13] Onishi N (2014). Clashes erupt as Liberia sets an Ebola quarantine, *The New York Times*.

[ref14] Rabelo I, Lee V, Fallah MP, Massaquoi M, Evlampidou I, Crestani R, Decroo T, Van den Bergh R, Severy N (2016). Psychological distress among Ebola survivors discharged from an Ebola Treatment Unit in Monrovia, Liberia – a qualitative study. Frontiers in Public Health 4, 1–7. 27458576 10.3389/fpubh.2016.00142 PMC4931229

[ref15] Van Bortel T, Basnayake A, Wurie F, Jambai M, Koroma AS, Muana AT, Hann K, Eaton J, Martina S, Nellumsa LB (2016). Psychosocial effects of an Ebola outbreak at individual, community and international levels. Bulletin of the World Health Organization 94, 210–214. 26966332 10.2471/BLT.15.158543 PMC4773931

[ref16] WHO (2016). Ebola data and statistics. http://apps.who.int/gho/data/view.ebola-sitrep.ebola-summary-20160511?lang=en.

[ref17] WHO Ebola Response Team (2016). After Ebola in West Africa—unpredictable risks, preventable epidemics. New England Journal of Medicine 375, 587–596. 27509108 10.1056/NEJMsr1513109

[ref18] World Bank (2016). 2014-2015 West Africa Ebola Crisis: Impact Update. Technical Report, World Bank. http://www.worldbank.org/en/topic/macroeconomics/publication/2014-2015-west-africa-ebola-crisis-impact-update.

