# Supplementary material for: The impact of the 2014 Ebola virus disease outbreak in Liberia on parent preferences for harsh discipline practices: a quasi-experimental, pre-post design
Source: Glob Ment Health (Camb). 2018 Jan 9;5:e1. doi: 10.1017/gmh.2017.24 (PMC5797936; doi:10.1017/gmh.2017.24)
Supplement: Supplementary file 1 [file S2054425117000243sup001.pdf]

AppendixA. Appendix

Table A.1: Assocations between EVD exposure and parent-reported Strengths & Difficulties Questionnaire for children, individual items & sub-scales

	EVD in Household	No EVD in Houshold	
	Adj. Mean	Adj. Mean	t-stat
**Total Difficulties Score**	0.77	0.92	2.22
**Conduct Problems Sub-scale**	0.61	0.81	1.82
Often loses temper	0.61	1.02	2.08
Generally well behaved, usually does what adults request	1.13	1.43	1.71
Often fights with other children or bullies them	0.66	1.10	2.24
Often argumentative with adults	0.46	0.55	0.51
Can be spiteful to others	0.47	0.82	1.84
**Emotional Symptoms Sub-scale**	0.86	0.94	0.74
Often complains of headaches, stomach-aches or sickness	0.83	0.94	0.53
Many worries or often seems worried	0.64	0.91	1.52
Often unhappy, depressed or tearful	1.13	0.95	0.96
Nervous or clingy in new situations, easily loses confidence	0.47	0.87	1.89
Many fears, easily scared	1.18	1.00	0.96
**Hyperactivity/Inattention Sub-scale**	1.09	1.10	0.12
Restless, overactive, cannot stay still for long	1.07	1.23	0.71
Constantly fidgeting or squirming	1.24	1.28	0.21
Easily distracted, concentration wanders	1.02	1.00	0.11
Can stop and think things out before acting	1.00	0.91	0.40
Good attention span, sees work through to the end	0.77	1.10	1.54
**Peer/Relationship Problems Sub-scale**	0.55	0.85	3.46
Rather solitary, prefers to play alone	0.64	0.81	0.79
Has at least one good friend	1.42	1.29	0.58
Generally liked by other children	1.85	1.41	2.81
Picked on or bullied by other children	0.78	1.18	2.07
Gets along better with adults than with other children	0.59	0.98	1.75
**Prosocial Sub-scale**	1.17	1.15	0.21
Considerate of other people's feelings	1.02	1.09	0.31
Shares readily with other children, for example toys, treats, pencils	1.28	1.32	0.22
Helpful if someone is hurt, upset or feeling ill	0.79	0.82	0.17
Kind to younger children	1.54	1.37	1.09
Often offers to help others (parents, teachers, other children)	1.13	1.03	0.50

Note. Answers to each item range between 0 and 2. Sub-scales are created by averaging responses for items in each category. Total Difficulties Score is used in the main analysis and excludes the prosocial sub-scale. Means are adjusted for child's gender and age at baseline.
